# Exacerbated Skeletal Muscle Phenotype in Mice with ‘Homotypic’ Expression of the Tubular Aggregate Myopathy ORAI1 G100S Mutation

**DOI:** 10.3390/biomedicines14030587

**Published:** 2026-03-05

**Authors:** Nan Zhao, Miao He, Robert T. Dirksen

**Affiliations:** Department of Pharmacology and Physiology, University of Rochester Medical Center, 601 Elmwood Avenue, Rochester, NY 14642, USA; nan_zhao@urmc.rochester.edu (N.Z.);

**Keywords:** skeletal muscle, calcium signaling, store operated calcium entry, ORAI1

## Abstract

**Background:** Tubular aggregate myopathy (TAM) is an autosomal dominant myopathy that results from gain-of-function mutations in the *STIM1* and *ORAI1* genes, which encode the two key proteins that coordinate store-operated Ca^2+^ entry in skeletal muscle and other cell types. Knock-in mice heterozygous for a glycine-to-serine point mutation in the ORAI1 pore (*ORAI1^G100S/+^* or GS mice) phenocopy several key aspects of TAM in humans with the analogous mutation including muscle weakness, exercise intolerance, elevated CK levels, hypocalcemia, and the presence of tubular aggregates. **Methods:** Since homozygous inheritance of the ORAI1-G100S mutation is embryonic lethal, we assessed the impact of homotypic ORAI1-G100S expression in skeletal muscle by crossing GS mice with constitutive, muscle-specific ORAI1 knock-in mice (cORAI1-KO). **Results:** Compound cORAI1-KO/GS mice exhibit only one active *ORAI1* (GS) allele, and thus only express ORAI1-G100S monomers in skeletal muscle (‘homotypic’ GS mice). Homotypic GS mice exhibit an earlier onset and more severe muscle phenotype than age-matched heterotypic GS mice with both WT and GS alleles. Specifically, homotypic GS mice exhibit TAs at an earlier age, as well as significantly reduced *in vivo* muscle performance (grip strength, treadmill endurance, and rotarod endurance), maximal specific force production, and respiratory function, compared to those observed for both WT and heterotypic GS mice. **Conclusions:** These findings indicate that homotypic expression of the ORAI1-G100S mutation in skeletal muscle results in an earlier-onset and more severe muscle phenotype.

## 1. Introduction

Calcium ions (Ca^2+^) serve as universal second messengers. The intracellular Ca^2+^ concentration in cells is maintained at a low level to prevent cell damage and ensure proper cellular function [1,2,3,4,5]. Elevated concentrations of intracellular Ca^2+^ are either removed from the cell by plasma membrane Ca^2+^ pumps/exchangers or sequestered in intracellular organelles, especially the endoplasmic/sarcoplasmic reticulum (ER/SR). The opening of Ca^2+^-selective ion channels in the plasma membrane and ER/SR allows Ca^2+^ to rapidly enter the cytosol and initiate downstream Ca^2+^ signaling cascades [6,7]. A crosstalk mechanism between the plasma membrane and ER/SR, known as store-operated calcium entry (SOCE), is activated in response to depletion of the ER/SR Ca^2+^ stores, allowing Ca^2+^ ions to enter the cell from the extracellular space [8]. Two key proteins coordinate the SOCE pathway: (1) STIM1, a luminal Ca^2+^ sensor protein located in the ER/SR membrane, and (2) ORAI1, a highly selective, Ca^2+^-permeable channel in the plasma membrane [3,9]. Upon ER/SR Ca^2+^ store depletion, unbinding of Ca^2+^ from the STIM1 luminal EF-hand motifs triggers STIM1 oligomerization and translocation to ER/SR–plasma membrane junctions [10,11]. STIM1 oligomers within these junctions interact with and open ORAI1 Ca^2+^ entry channels, facilitating Ca^2+^ influx from the extracellular space needed to refill ER/SR Ca^2+^ stores [12,13,14,15]. In skeletal muscle, STIM1-ORAI1 signaling plays a key role in both skeletal muscle development [16,17,18] and fatigue resistance [19,20,21,22,23].

Mutations in STIM1 and ORAI1 result in both loss-of-function and gain-of-function genetic disorders. Recessive loss-of-function STIM1/ORAI1 variants impair SOCE and lead to severe combined immunodeficiency (SCID) [3,9,24]. On the other hand, tubular aggregate myopathy (TAM) [25,26,27,28,29,30] is caused by dominant gain-of-function mutations in STIM1 and ORAI1 that result in constitutive Ca^2+^ influx, independent of ER/SR store depletion [9,31]. TAM is part of a broader clinical spectrum that forms a continuum with Stormorken syndrome (STRMK; OMIM #160565 and #615883), in which disease severity and pathological features vary depending on the affected gene and the specific mutation [32,33,34,35].

Despite the genetic heterogeneity of TAM, skeletal muscle structure and function are consistently impacted, with clinical manifestations ranging from only asymptomatic elevations in creatine kinase (CK) to a significant progressive, childhood-onset muscle weakness, particularly affecting upper and lower limbs, that is accompanied by exercise intolerance, muscle pain, cramping, and myalgia [31,36]. A consistent histological feature observed in muscle biopsies from TAM patients is the presence of tubular aggregates (TAs) [25,26,27,28,29,36]. TAs are abnormal accumulations of highly ordered and tightly packed tubules within skeletal muscle fibers that appear as honeycomb-like structures in EM images [36,37]. Several studies indicate that TAs are formed by membranes of SR origin, though the precise molecular mechanisms that underlie TA formation and expansion remain unclear [26,27,28,29,36,38]. Additional multi-systemic manifestations, including thrombocytopenia, ichthyosis, anemia, and miosis, are also observed, particularly in TAM resulting from mutations in STIM1 [25,26,27,28,29]. Missense mutations in genes encoding calsequestrin (*CASQ1*), a muscle-specific SR Ca^2+^-binding protein, and the type 1 ryanodine receptor SR Ca^2+^-release channel (*RYR1*), have also been associated with TAM, though these cases typically present with a milder phenotype characterized by adult-onset muscle weakness in the absence of multi-systemic involvement [39,40,41].

Recently, we generated a mouse model of TAM resulting from a glycine-to-serine point mutation in the ORAI1 pore region (*ORAI1^G100S/+^* ‘heterozygous’ GS mice) [42]. These mice are heterozygous for the G100S mutation in ORAI1, which corresponds to the human *ORAI1^G98S/+^* mutation identified in at least three unrelated families worldwide [28,29]. The G100S mutation impairs pore closure, and thus leads to constitutive, STIM1-independent Ca^2+^ entry [43]. Heterozygous GS mice recapitulate several key clinical and histopathological features of TAM observed in humans with the analogous ORAI1-G98S variant, including elevated CK, hypocalcemia, muscle weakness, exercise intolerance, and the presence of TAs [42]. Moreover, heterozygous GS mice do not exhibit significant hematological dysfunction or changes in platelets, red blood cells, or bleeding/clotting function [42], consistent with clinical observations in the majority of *ORAI1^G98S/+^* patients (only one of six G98S TAM patients exhibited anemia and thrombocytopenia was not observed in any of these individuals) [28,29].

Interestingly, we found that interbreeding heterozygous GS mice did not result in any viable homozygous offspring from nine confirmed pregnancies [42]. Moreover, no homozygous mice were observed among another 35 embryos (E14.5–E16.5) collected from five crosses of heterozygous GS mice. Thus, homozygous inheritance of the *G100S* allele results in early embryonic lethality. These findings suggest that homotypic G100S expression (expression of G100S-ORAI1 in the absence of WT-ORAI1) results in a more severe disease phenotype.

To further investigate the biological impact of homotypic GS expression on skeletal muscle, we generated constitutive, skeletal muscle-specific homotypic GS mice (*ORAI1^fl/G100S:MCK-Cre^* mice) by crossing heterozygous GS mice with constitutive, muscle-specific *ORAI1* knockout mice (*ORAI1^fl/fl:MCK-Cre^* mice) [22,44]. The resulting offspring include mice that carry one functional *ORAI1* allele (either wild-type or *G100S*) and one null *ORAI1* allele in skeletal muscle. Using this approach, we generated viable mice in which skeletal muscle exhibited ‘homotypic’ expression of the G100S-ORAI1 variant (*ORAI1^fl/G100S:MCK-Cre^* or ‘homotypic’ GS mice). Notably, we found that constitutive, homotypic GS mice exhibit a more severe and earlier-onset skeletal muscle phenotype (e.g., impaired muscle strength and exercise tolerance) compared to that reported for age-matched heterotypic GS (*ORAI1^fl/+:MCK-Cre^*) mice. In addition, while the muscle phenotypes of heterotypic GS mice are restricted to limb muscles, we found that homotypic GS mice also exhibit evidence of significantly impaired respiratory function, consistent with diaphragm and/or intercostal muscle dysfunction. These findings indicate that homotypic GS expression in muscle results in an earlier-onset and broader impact on skeletal muscle function compared to that observed for heterotypic GS mice.

## 2. Methods

### 2.1. Animals

All animal procedures were conducted in accordance with institutional guidelines approved by the University Committee on Animal Recourses at the University of Rochester Medical Center. Mice were housed under standard conditions with *ad libitum* access to water and standard chow under a regulated ambient temperature and on a 12:12 light/dark cycle.

The generation of floxed ORAI1 mice (ORAI1^tm1.2Hjm^ or *ORAI1^fl/fl^* mice) was described previously [45]. These mice were crossed with Muscle Creatine Kinase (MCK)-Cre mice (Jackson Laboratory strain 006475; The Jackson Laboratory, ME), then subsequently back-crossed with *ORAI1^fl/fl^* mice. This led to the generation of muscle specific, constitutive ORAI1 knockout (*ORAI1^fl/fl:MCK-Cre^*) mice. The breeding scheme used in this study involved crossing *ORAI1^fl/fl:MCK-Cre^* mice with heterozygous male *ORAI1^G100S/+^* (GS) mice as described previously [42]. The following four genotypes were generated: *ORAI1^fl/+^* (cfl/+), *ORAI1^fl/G100S^* (cfl/GS), *ORAI1^fl/+:MCK-Cre^* (cORAI1-KO/+), and *ORAI1^fl/G100S:MCK-Cr^* (cORAI1-KO/GS). Since the heterozygous GS mice are on a C57BL6/J background while the *ORAI1^fl/fl:MCK-Cre^* mice are on a C57BL6/N background, the littermate mice used in this study were on a mixed C57BL6/N and J background. Genotyping primers are summarized in Table 1.

Inducible ORAI1 knockout (*ORAI1^fl/fl:HSA-MCM^* mice) mice were generated by crossing *ORAI1^fl/fl^* mice with muscle-specific, inducible Human Skeletal Actin (HSA)-mutated estrogen receptor (Mer)-Cre-Mer (MCM) mice on a C57Bl/6J background [46]. *ORAI1^fl/fl:HSA-MCM^* mice were backcrossed with *ORAI1^fl/fl^* mice and maintained on a mixed C57BL/6N and J background. The breeding scheme used in these studies involved crossing female *ORAI1^fl/fl:HSA-MCM^* mice with heterozygous male GS mice, resulting in *ORAI1^G100S/fl^* (ifl/GS) and *ORAI1^G100S/fl:HSA-MCM^* (iORAI1-KO/GS) littermates. Genotyping primers are summarized in Table 1.

### 2.2. Tamoxifen Preparation and Administration

Tamoxifen (Sigma Aldrich, St. Louis, MO, USA) was dissolved in corn oil at a concentration of 20 mg/mL by overnight shaking at room temperature. The dissolved tamoxifen was aliquoted, stored protected from light at 4 °C, and used within one month of preparation. Six-month-old (6M) ifl/GS and iORAI1-KO/GS mice received intraperitoneal (i.p.) injections of tamoxifen at a dose of 40 mg/kg every 24 h for five consecutive days. One week later, the same injection regimen was repeated for another five days. Terminal experiments were conducted once the mice reached ~8 months of age (2 months after initial tamoxifen administration).

### 2.3. Quantitative RT-PCR (RT-qPCR)

Total RNA from snap-frozen tibialis anterior muscles was isolated using Trizol reagent (Thermo Fisher Scientific, Waltham, MA, USA) according to the manufacturer’s protocol, then purified using an RNAse mini kit (Qiagen, Hilden, Germany). RNA (1 µg) was mixed with DNase (Life Technologies, Carlsbad, CA, USA), Super Script III (Life Technologies, Carlsbad, CA, USA), dNTPs (Life Technologies, Carlsbad, CA, USA), and oligo(dT) (Life Technologies, Carlsbad, CA, USA) as previously described [47]. RT-qPCR was performed on a Step One Plus Real-Time PCR machine (Applied Biosystems, Waltham, MA, USA) using SYBR Green FastMix (Quantabio, Beverly, MA, USA). *GAPDH* was—used as a loading control. Primer sequences are summarized in Table 2. *ORAI1* primers were designed using regions that were not altered by the edited G100S mutation. mRNA levels were analyzed using the 2^−∆∆CT^ method as previously described [48].

### 2.4. Mn^2+^ Quench Measurements

Acutely dissociated flexor digitorum brevis (FDB) fibers were loaded with 5 μM fura-2 AM (Thermo Fisher Scientific, Waltham, MA, USA) in Ca^2+^-free Ringer’s solution (145 mM NaCl, 5 mM KCl, 3 mM MgCl_2_, and 0.2 mM EGTA, pH 7.4) for 1 h at 37 °C. A skeletal-muscle myosin inhibitor (30 μM N-benzyl-*p*-toluene sulfonamide [BTS]) was included, along with fura-2 AM, to limit musclefiber movement. In experiments measuring maximal SOCE activity, fibers were pre-incubated with a SERCA pump inhibitor cocktail (1 μM thapsigargin [TG] and 15 μM cyclopiazonic acid [CPA]) in addition to BTS and fura-2 AM. For measurements of both constitutive Ca^2+^ entry (no store depletion) and SOCE (after store depletion), FDB fibers were first equilibrated in Ca^2+^-free Ringer’s solution at the end of the incubation period, and then excited at the fura-2 isosbestic point and emission was detected at 510 nm using a DeltaRam illumination system (Photon Technology International, Lawrenceville, NJ, USA). After obtaining an initial baseline rate of fura-2 fluorescence decay, fibers were exposed to Ca^2+^-free Ringer’s solution supplemented with 0.5 mM MnCl_2_. The maximum rate of Ca^2+^ entry was calculated as the peak differential of the fura-2 emission during Mn^2+^ application (dF/dt in counts/sec) as described previously [42].

### 2.5. Cryopreservation

Extensor digitorum longus (EDL) muscles excised from 4M cfl/+, cfl/GS, cORAI1-KO/+, and cORAI1-KO/GS mice were immediately incubated in 30% sucrose solution for 8–24 h at 4 °C and then embedded with optimal cutting temperature compound (OCT) (ThermoFisher, Waltham, MA, USA). Embedded muscles were then flash frozen in isopentane at −55 °C and stored at −80 °C until use. Prior to staining (see below), 5 μm or 10 μm thick cross sections of flash frozen EDL muscles were cut and mounted onto Superfrost Plus slides (ThermoFisher, Waltham, MA, USA) using a Leica cryostat (Leica Microsystems, Wetzlar, Germany) at −20 °C.

### 2.6. Gomori Trichrome Staining

Sections (5 μm) of EDL muscles from 4M cfl/+, cfl/GS, cORAI1-KO/+, and cORAI1-KO/GS mice were stained with Gomori trichrome as described previously [42]. Images were captured using a Keyence BZ-X800 epifluorescence microscope (Keyence, Osaka, Japan) at 40× (0.6-Air Plano Apochromat).

### 2.7. Fiber Typing

Cross sections (10 μm) of EDL muscles from 4M cfl/+, cfl/GS, cORAI1-KO/+, and cORAI1-KO/GS mice were blocked for 30 min in blocking buffer (0.2% Triton X-100, 10% goat serum in phosphate-buffered saline [PBS] solution), then incubated overnight at 4 °C with BA-DA [1:40, myosin heavy chain type I antibody] (DSHB, Iowa City, IA, USA), SC-71 [1:40, myosin heavy chain type IIa antibody] (DSHB, Iowa City, IA, USA), BF-F3 [1:40, myosin heavy chain type IIb antibody] (DSHB, Iowa City, IA, USA), and rabbit anti-laminin [1:500] (Sigma Aldrich, St. Louis, MO, USA) antibodies. The following day, samples were washed with PBS and incubated with secondary antibodies for 1 h at room temperature. The following secondary antibodies were used: Goat anti-Mouse IgG1, Alexa Fluor™ 555 [1:1000] (Life Technologies, Carlsbad, CA, USA), Goat anti-Mouse IgM, Alexa Fluor™ 488 [1:1500] (Life Technologies, Carlsbad, CA, USA), Goat anti-Mouse IgG2b, Alexa Fluor™ 350 [1:1500] (Life Technologies, Carlsbad, CA, USA), and Goat anti-Rabbit IgG, Alexa Flour^®^ 647 [1:1500] (Life Technologies, Carlsbad, CA, USA). Muscle sections were mounted with Fluoromount-G mounting media (Southern Biotech, Birmingham, AL, USA) and dried in the dark at room temperature. Images were captured using a Keyence BZ-X800 epifluorescence microscope (Keyence, Osaka, Japan) at 4× (0.10-Air Plano Apochromat). The cross sectional area (CSA) and fiber type distribution were analyzed using the SMASH software [49].

### 2.8. Wire Hang Test

Mice were placed on a metal grid, and after once having established a firm grip, the grid was inverted 10–12 inches above a soft, padded surface. The latency to fall was measured over three consecutive trials, with a 10-min rest period between each trial. The experiment was terminated if mice stayed on the grid for over 60 s. The fractional reduction in hanging time was calculated as the difference in latency between the first (T1) and third trial (T3) divided by the latency of the first trial: (T1-T3)/T1.

### 2.9. Treadmill Endurance Test

Mice first underwent a 3-day acclimation training session on a treadmill (Columbus Instruments, Columbus, OH, USA) run at 5 m/min on a 15° incline. On the fourth day, mice were placed on the treadmill with an initial 5-min warmup phase at 5 m/min and 15° incline, followed by a gradual increase in speed of 1 m/min every minute until reaching a maximum speed of 20 m/min. This speed was then maintained for the remainder of the experiment. The entire treadmill endurance protocol lasted 60 min and covered a total distance of 1 km. To ensure continuous running, if a mouse stopped, a brief burst of air (lasting less than 1 s) was delivered to its backside using a Whoosh Duster™, and the pause was counted as a 3 s rest. The total running distance and a completion curve, recording the number of mice remaining on the treadmill at each minute for each group, were plotted and compared.

### 2.10. Rotarod Endurance Test

A 3-day acclimation training session for 20 min at 15 revolution per minute (rpm) on a Rotamex-5 (Columbus Instruments, Columbus, OH, USA) was performed prior to the experimental session, as described previously [21]. On the following (fourth) day, trained mice were placed on the rotarod at an initial speed of 14 rpm with an increment of 1 rpm every 5 min until reaching a maximum speed of 23 rpm. This speed was then maintained until the 60 min protocol was completed. After each fall, the mouse was promptly placed back on the rotarod, and a 3 s rest was recorded. The total running distance (revolutions) and a completion curve, recording the number of mice remaining on the treadmill at each minute for each group, were plotted and compared.

### 2.11. Whole Body Plethysmography

Respiratory function was assessed using Buxco Small Animal Whole Body Plethysmography Chambers (Buxco Research Systems, Wilmington, NC, USA). Mice were first acclimated to the system over two consecutive days, and experimental data were collected on the third day. On each of the three days, a single conscious, unrestrained mouse was placed in the chamber for a 15-min acclimation period, followed by 10 min of data acquisition on day 3 [50]. Respiratory metrics were calculated using Fine Point software version 2.8.0.12146 (Buxco Research Systems, Wilmington, NC, USA) and included breathing frequency (BPM), mid-tidal expiratory flow (EF50), and peak inspiratory/expiratory flow (PiFb/PeFb).

### 2.12. Ex Vivo Muscle Contractility

First, 4M cfl/+, cfl/GS, cORAI1-KO/+ and cORAI1-KO/GS mice were anesthetized by i.p. injection of 100 mg/kg ketamine, 10 mg/kg xylazine and 3 mg/kg acepromazine. EDL muscles were excised and mounted between two platinum electrodes in oxygenated Ringer solution (137 mM NaCl, 5 mM KCl, 1.2 mM NaH_2_PO_4_, 1 mM MgSO_4_, 2 mM CaCl_2_, 10 mM glucose, and 24 mM NaHCO_3_, pH 7.4) at 30 °C with one end connected to a 300C-LR dual mode force transducer (Aurora Scientific, Aurora, ON, Canada). The optimal muscle length was determined using a series of twitches stimulated with an ASI muscle contraction system equipped with a 701C stimulator (Aurora Scientific, Aurora, ON, Canada). Stimulus output was set at 120% of the required voltage to elicit maximal muscle force. Muscles were then equilibrated using three tetani (500 ms, 150 Hz) with 1 min intervals. A standard force frequency protocol (from 1 Hz to 250 Hz) was then used to determine the frequency dependence of muscle force production. After a 5-min rest period, muscles were stimulated with a repetitive moderate-frequency protocol (60 consecutive, 500 ms duration, 50 Hz stimulus trains delivered every 2.5 s). Muscle force was recorded and analyzed using DMC/DMA-HT software (Aurora Scientific, Canada). The physiological CSA and specific force were calculated for each muscle as described previously [51].

### 2.13. Statistical Analyses

Experimenters were blinded to genotype when conducting all behavioral assays (wire hang, rotarod endurance, and treadmill endurance tests), plethysmography, and immunofluorescence staining. Results are reported as mean ± SEM (standard error of mean). For continuous data, Shapiro-Wilk test and F tests were performed before statistical analysis to check for normality and homoscedasticity. For comparisons between two groups, Student’s *t*-test was performed. For experiments with 3 or more groups, statistical significance was determined using one-way analysis of variance (ANOVA), followed by Turkey’s *post hoc* test for multiple comparisons. GraphPad Prism 7 (GraphPad Software Inc., San Diego, CA, USA) was used for all statistical analyses and to graph plots. *p* < 0.05 was considered statistically significant. Individual data points in each bar graph reflect data obtained from a different mouse.

## 3. Results

### 3.1. Generation of Mice with Homotypic Expression of ORAI1-G100S in Skeletal Muscle

Five different murine models of TAM resulting from gain-of-function mutations in either STIM1 or ORAI1 were described previously [42,52,53,54,55]. None of these TAM models (nor TAM patients) exhibit a homozygous inheritance pattern. Moreover, we previously reported that homozygous inheritance of the GS allele (*ORAI1^G100S/G100S^*) was embryonic lethal [42]. Specifically, from multiple intercrosses of heterozygous *ORAI1^G100S/+^* mice, no homozygous *ORAI1^G100S/G100S^* mice were obtained among 35 embryos collected at embryonic days 14.5–16.5.

To overcome this limitation and assess the impact of homotypic expression of ORAI1-G100S channels on skeletal muscle, we generated constitutive homotypic GS mice. Due to the maternal cannibalization of heterozygous GS mice reported earlier [42], heterozygous male GS mice were bred with female muscle-specific, constitutive ORAI1-KO (*ORAI1^fl/fl:MCK-Cre^*) mice. This breeding scheme resulted in the following two relevant compound heterozygous offspring: (1) control *ORAI1^+/fl:MCK-Cre^* mice (cORAI1-KO/+ mice) and (2) homotypic *ORAI1^GS/fl:MCK-Cre^* GS mice (cORAI1-KO/GS mice). The skeletal muscle of both genotypes only possesses a single (homotypic) functional *ORAI1* allele—either WT ORAI1 (cORAI1-KO/+) or G100S ORAI1 (cORAI1-KO/GS). The phenotypes of these mice were compared to their Cre-negative littermate controls (*ORAI1^+/fl^* or cfl/+ mice; *ORAI1^GS/fl^* or cfl/GS mice), which each possesses a second functional floxed WT *ORAI1* allele.

**Figure 1:**
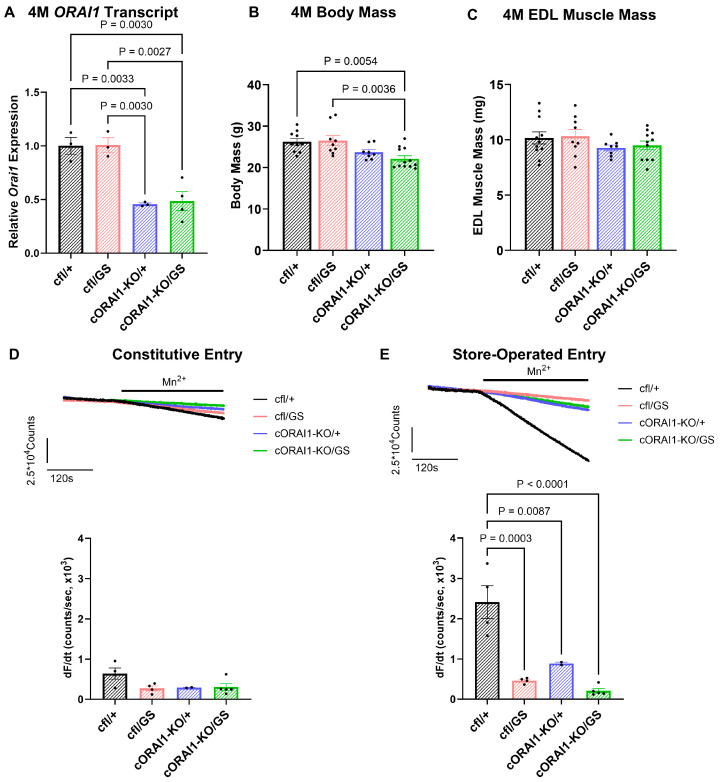
Phenotypic characterization of 4M homotypic cORAI1-KO/GS mice. (**A**) Average (±SEM) *ORAI1* transcript levels normalized to *GAPDH* as a reference gene in tibialis anterior muscles from 4M cfl/+, cfl/GS, cORAI1-KO/+ and cORAI1-KO/GS mice using RT-qPCR. (**B**,**C**) Average (±SEM) body mass (**B**) and EDL muscle mass (**C**) of 4M cfl/+, cfl/GS, cORAI1-KO/+ and cORAI1-KO/GS mice. (**D**) Representative traces of constitutive entry (*upper*) and average (±SEM) peak maximum rate of constitutive entry (*lower*) in FDB fibers from 4M cfl/+, cfl/GS, cORAI1-KO/+ and cORAI1-KO/GS mice. (**E**) Representative traces of store-operated entry (*upper*) and average (±SEM) peak maximum rate of store-operated entry (*lower*) in FDB fibers from 4M cfl/+, cfl/GS, cORAI1-KO/+ and cORAI1-KO/GS mice. Significance was calculated using a 1-way ANOVA test followed by Tukey’s multiple-comparisons post hoc test. Exact *p*-values where *p* < 0.05 are provided in the figure.

Successful knockout of one *ORAI1* allele in cORAI1-KO/GS and cORAI1-KO/+ mice was confirmed by RT-qPCR. At 4M, tibialis anterior muscle from both cORAI1-KO/GS and cORAI1-KO/+ mice showed ~50% reduction in total *ORAI1* transcripts (Figure 1A), consistent with efficient deletion of one *ORAI1* allele and a lack of compensatory upregulation from the other allele. Notably, cORAI1-KO/GS (homotypic GS) mice exhibited a significantly reduced body weight compared to their Cre-negative (cfl/GS or heterotypic GS) counterpart (Figure 1B). Despite this difference in body weight, all four groups exhibited comparable EDL muscle mass (Figure 1C), indicating that muscle size was unaffected by genotype at this age. However, it is unclear if the reduction in body mass of cORAI1-KO/GS mice is due to changes in the mass of larger muscles, fat mass, or other tissues.

We previously reported that SOCE in skeletal muscle fibers of GS mice is reduced across all ages. On the other hand, constitutive Ca^2+^ entry, while increased during early development, is abolished during postnatal development in part due to a reduction in ORAI1 protein expression [42]. Here, we quantified constitutive and store-operated Ca^2+^ entry in acutely dissociated FDB fibers from 4M cfl/+, cfl/GS, cORAI1-KO/+, and cORAI1-KO/GS mice (Figure 1D,E). 

For these studies, we used the maximum rate of Mn^2+^ quench of fura-2 fluorescence in FDB fibers under control (non-store depletion) conditions as an index of constitutive Ca^2+^ entry. In addition, the maximum rate of Mn^2+^ quench of fura-2 fluorescence recorded following SR Ca^2+^ store depletion (prior incubation with 1 μM thapsigargin + 15 μM cyclopiazonic acid) was used as an index of SOCE. Similar to that reported previously [42], constitutive entry was essentially absent in FDB fibers from 4M WT (cf/+) and heterotypic GS (cfl/GS) mice, as well as in FDB fibers from 4M cORAI1-KO/+ and cORAI1-KO/GS mice (Figure 1D). Thus, the presence of gain-of-function ORAI1-G100S subunits did not result in constitutive entry in adult FDB fibers in either the presence or absence of a viable WT ORAI1 allele. Interestingly, SOCE was markedly reduced in FDB fibers from 4M cORAI1-KO/+, cfl/GS, and cORAI1-KO/GS mice compared to that observed for FDB fibers from 4M WT mice (Figure 1E). These findings indicate that the compensatory mechanisms responsible for inhibiting constitutive and store-operated Ca^2+^ entry through G100S-containing ORAI1 channels in adult skeletal muscle were similar for both heterotypic (cfl/GS) and homotypic (cORAI1-KO/GS) GS expression.

### 3.2. Homotypic ORAI1-G100S Mice Exhibit Earlier Onset of Muscle Weakness and Exercise Intolerance

**Figure 2:**
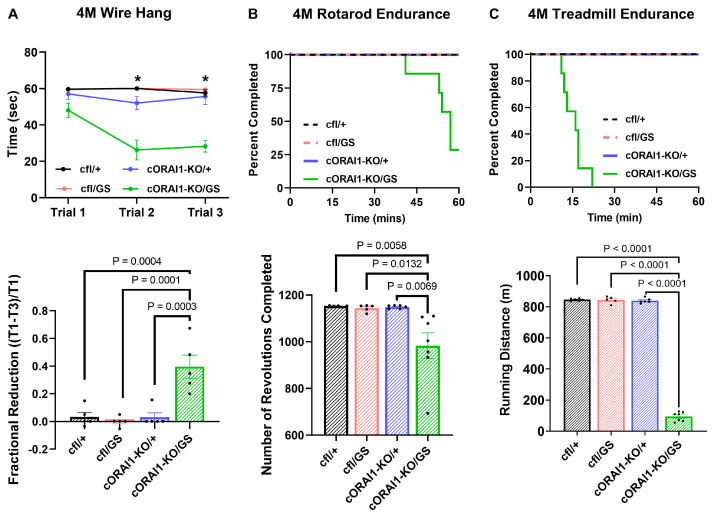
Homotypic cORAI1-KO/GS mice exhibit muscle weakness and exercise intolerance. (**A**) Wire-hang test of 4M cfl/+, cfl/GS, cORAI1-KO/+, and cORAI1-KO/GS mice where the time mice remain hanging on the grip were plotted (*upper*). The fractional reduction in hanging time was calculated as the difference in latency between the first grip (T1) and the third grip (T3), divided by the latency of the first grip: (T1-T3)/T1 (*lower*). (**B**,**C**) Rotarod endurance (**B**) and treadmill endurance (**C**) tests in 4M cfl/+, cfl/GS, cORAI1-KO/+, and cORAI1-KO/GS mice where the fraction of mice remaining on the apparatus during experiment (*upper*) and the total running distance (*lower*) were plotted. Significance was assessed using a two-way ANOVA (**A**) or one-way ANOVA (**B**,**C**), followed by Tukey’s multiple-comparisons post hoc test. * indicates *p* < 0.05 for cORAI1-KO/GS vs. cfl/+, cfl/GS and cORAI1-KO/+ mice. For bar graphs, the exact *p*-values are shown where *p* < 0.05.

We next assessed the impact of constitutive homotypic GS expression on skeletal muscle function in a series of *in vivo* behavioral studies. In the first trial of the wire hang test, all four groups exhibited similar hanging times (Figure 2A *upper*). 

However, unlike the control genotypes (cfl/+, cfl/GS, and cORAI1-KO/+ mice), hanging time decreased significantly for 4M homotypic cORAI1-KO/GS mice during the second and third trials, despite sufficient rest time (10 min) between each trial (Figure 2A *upper*) (*p* < 0.0001 for all conditions). Consequently, compared to all other groups, the fractional hanging time reduction of cORAI1-KO/GS mice was significantly increased, consistent with enhanced muscle fatigue (Figure 2A *lower*). Mice were also assessed for exercise tolerance using both rotarod and treadmill endurance tests (Figure 2B and Figure 2C, respectively). Compared to the three control genotypes where every mouse in the group successfully completed both tasks, only two out of seven cORAI1-KO/GS mice completed the rotarod endurance task, and none of these mice completed the treadmill endurance task (Figure 2B,C, *upper*). Moreover, total running distance was significantly reduced for cORAI1-KO/GS mice in both the rotarod and treadmill endurance tasks (Figure 2B,C, *lower*). Results in Figure 2 indicate that 4M homotypic GS mice exhibit greater muscle weakness and exercise intolerance compared to age-matched heterotypic GS (cfl/GS) mice, supporting the idea that homotypic G100S expression results in a more severe and earlier-onset disease phenotype than heterotypic G100S expression.

### 3.3. Homotypic ORAI1-G100S Mice Exhibit Compromised Ventilatory Function

**Figure 3:**
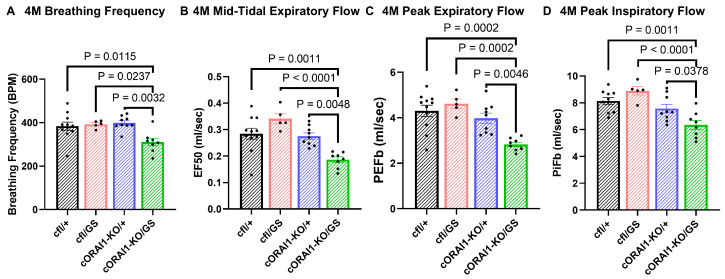
Homotypic cORAI1-KO/GS mice exhibit reduced ventilatory function. 4M cORAI1-KO/GS mice exhibited significantly reduced breathing frequency (**A**), mid-tidal expiratory flow (EF50) (**B**), peak expiratory flow (PeFb) (**C**), and peak inspiratory flow (PiFb) (**D**). Significance was determined using 1-way ANOVA followed by Tukey’s multiple-comparisons post hoc test. Exact *p*-values where *p* < 0.05 are shown.

Ventilatory function is not typically impaired in TAM patients and has not been previously reported in TAM mouse models. However, since our behavioral studies indicate an earlier onset and more severe degree of muscle dysfunction in homotypic GS mice, we compared the ventilatory function of 4M cfl/+, cfl/GS, cORAI1-KO/+, and cORAI1-KO/GS mice. In these studies, diaphragm and intercostal muscle function was indirectly assessed in conscious, ambulatory mice by measuring respiratory function using whole-body plethysmography (Figure 3). 

We found that breaths per minute (BPM, Figure 3A), mid-tidal expiratory flow (EF50, Figure 3B), peak expiratory flow (PeFb, Figure 3C), and peak inspiratory flow (PiFb) (Figure 3D) were all significantly reduced in 4M cORAI1-KO/GS mice compared to all other genotypes. These results indicate that ventilatory function is significantly reduced in 4M homotypic GS mice.

### 3.4. Homotypic ORAI1-G100S Mice Exhibit More Severely Reduced Ex Vivo Specific Force Production

**Figure 4:**
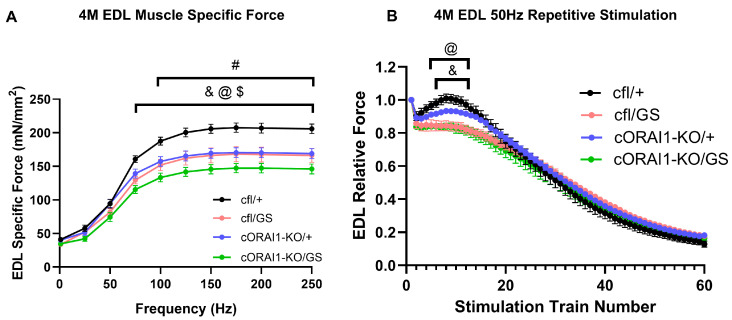
Homotypic cORAI1-KO/GS mice exhibit reduced *ex vivo* EDL peak force production. Average (±SEM) specific force-frequency curves (**A**) and peak specific force production during repetitive, high-frequency stimulation (60 trains of 50 H for 500 ms/train delivered every 2.5 s) (**B**) in EDL muscles from 4M cfl/+, cfl/GS, cORAI1-KO/+, and cORAI1-KO/GS mice. Significance was determined using 2-way ANOVA followed by Tukey’s multiple-comparisons post hoc test. & indicates *p* < 0.05 for cfl/+ vs. cfl/GS mice; @ indicates *p* < 0.05 for cfl/+ vs. cORAI1-KO/GS mice; $ indicates *p* < 0.05 for cORAI1-KO/+ vs. cORAI1-KO/GS mice; # indicates *p* < 0.05 for cfl/+ vs. cORAI1-KO/+ mice. N = 4, 4, 6, 5 for cfl/+, cfl/GS, cORAI1-KO/+ and cORAI1-KO/GS respectively.

We directly quantified skeletal muscle force production from *ex vivo* contractile force measurements in EDL muscles excised from 4M cfl/+, cfl/GS, cORAI1-KO/+, and cORAI1-KO/GS mice (Figure 4). Compared to 4M cfl/+ (WT) control mice, EDL muscles from all three of the other genotypes exhibited significantly reduced peak specific force production during stimulation at high frequencies (≥75 Hz for cfl/GS and cORAI1-KO/GS mice; ≥100 Hz for cORAI1-KO/+ mice) (Figure 4A). Notably, EDL muscles from cORAI1-KO/GS mice (Figure 4A, *green trace*) exhibited significantly reduced peak specific force compared to that of cORAI1-KO/+ and cfl/+ mice at ≥75 Hz. A similar trend—though not statistically significant—was observed for EDL muscles from homotypic GS (cORAI1-KO/GS) mice compared to heterotypic GS (cfl/GS) mice at ≥75 Hz (*p* = 0.298–0.147).

To evaluate specific force production during sustained activity, EDL muscles were also subjected to a repetitive, high-frequency stimulation protocol consisting of 60 stimulations under at 50 Hz stimulation (500 ms duration, every 2.5 s with a 0.2 duty cycle) (Figure 4B). As previously described [23,56], EDL muscles from the WT (cfl/+) mice displayed a rebound increase in specific force over several stimulus trains following the second stimulation train (Figure 4B, *black trace*), a phenomenon associated with enhanced SOCE activity. Consistent with that reported previously for 8M GS mice [42], this transient force augmentation was markedly blunted in EDL muscles from both 4M heterotypic (Figure 4B, *pink trace*) and homotypic GS mice (Figure 4B, *green trace*). EDL muscles from cORAI1-KO/+ mice displayed a modestly reduced rebound phase, but the difference relative to that of WT (cfl/+) control mice was not statistically significant (Figure 4B, *violet trace*). 

### 3.5. Histological Alterations in Skeletal Muscle of Homotypic ORAI1-G100S Mice

**Figure 5:**
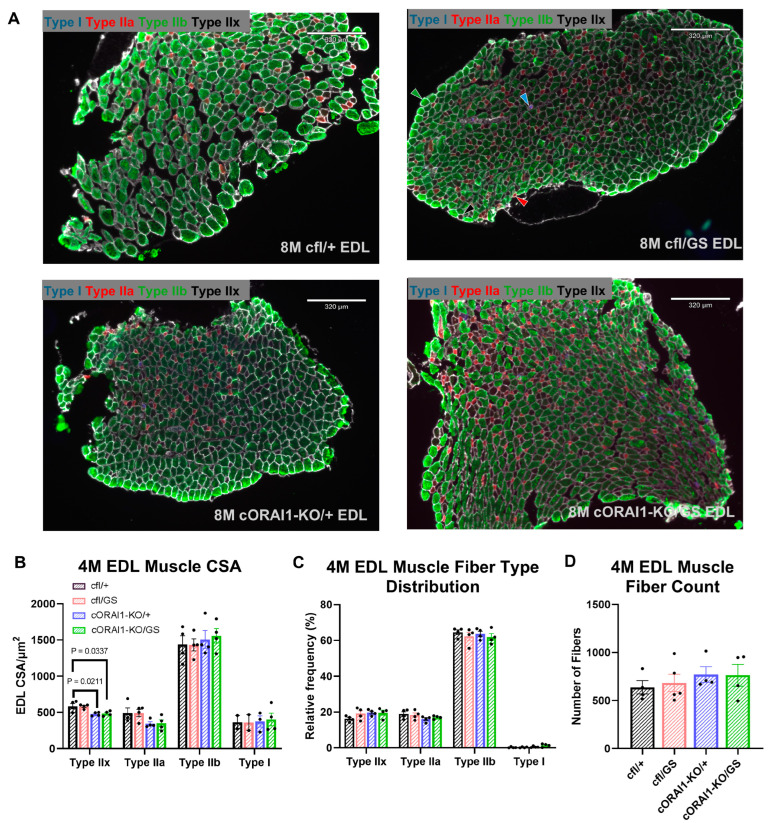
Fiber-type analysis of homotypic cORAI1-KO/GS mice. (**A**) Representative fiber type immunohistochemistry staining images of EDL muscles from 4M cfl/+, cfl/GS, cORAI1-KO/+, and cORAI1-KO/GS mice. Examples of each fiber type are indicated with the appropriate color-coded arrowhead (Type I, cyan; Type IIa, red; Type IIb, green; Type IIx, black) in the cfl/GS image *(upper right*). (**B**,**C**) Average (±SEM) cross-sectional area (CSA) (**B**), relative fiber type distribution (**C**) of different muscle fiber types and (**D**) number of fibers in EDL muscles of 4M cfl/+, cfl/GS, cORAI1-KO/+, and cORAI1-KO/GS mice. Significance was determined using 1-way ANOVA followed by Tukey’s multiple-comparisons post hoc test. Exact *p*-values where *p* < 0.05 are shown.

Increased fiber size variability and type I fiber predominance are histopathological features commonly observed in muscle biopsies from TAM patients [31,57,58]. However, these features are absent in muscle from GS mice [42]. To determine if homotypic GS expression recapitulates the fiber size variability and type I fiber predominance reported in TAM patients, we quantified the fiber type content and CSA of EDL muscle fibers from 4M cfl/+, cfl/GS, cORAI1-KO/+, and cORAI1-KO/GS mice (Figure 5). No changes in the relative proportion of different fiber types (type I, IIa, IIb, and IIx fibers) were observed across any of the genotypes (Figure 5C). Interestingly, constitutive, muscle-specific knockout of a single *ORAI1* allele (i.e., both cORAI1-KO/+ and cORAI1-KO/GS mice) resulted in a modest reduction in the CSA of type IIx fibers (Figure 5B), which was previously reported for EDL muscles from mice with constitutive, muscle-specific knockout of both *ORAI1* alleles [22]. similar trend was observed for type IIa fibers from cORAI1-KO/+ and cORAI1-KO/GS mice, although this was not statistically significant.

Previous studies utilized Gomori Trichrome staining to visualize TAs in muscle cross sections, which appear as distinct pink/purple inclusions [28,29,42]. Using this approach, no inclusions were detected in EDL muscle cross sections from 4M cfl/+, cfl/GS, or cORAI1-KO/+ mice (Supplemental Figure S1A–C), which is consistent with previous reports that TAs are observed in skeletal muscle of heterotypic GS mice beginning at 8M [42,59]. In contrast, internal pink/purple inclusions representing TAs were observed in EDL muscle from 4M cORAI1-KO/GS mice (Supplemental Figure S1D). Taken together, these findings suggest that homotypic expression of ORAI1-G100S channels accelerates the timeline for the formation of TAs, effectively reducing the onset time from 8M to ≤4M.

### 3.6. Inducible Homotypic ORAI1-KO/GS Mice Exhibit Intermediate Alterations in Muscle Function

To address potential compensatory changes due to early, constitutive homotypic GS expression in skeletal muscle in cORAI1-KO/GS mice, we also evaluated inducible, muscle-specific homotypic GS mice (iORAI1-KO/GS mice). iORAI1-KO/GS mice were generated by intercrossing heterozygous male GS mice with female inducible, muscle-specific ORAI1-KO mice (*ORAI1^fl/fl:HSA-Cre^* mice). For iORAI1-KO/GS mice, one *ORAI1* allele is flanked by loxP sites and modified estrogen-receptor domains fused to the N- and C-termini of Cre recombinase driven by the human skeletal muscle actin promoter (HSA-MCM) [46]. Thus, in these mice, tamoxifen administration triggers Cre-mediated deletion of the floxed *ORAI1* allele specifically in skeletal muscle. For these studies, ifl/GS mice (Cre-GS mice with a floxed *ORAI1* allele) were used as tamoxifen controls, as tamoxifen administration does not lead to the excision of the *ORAI1* allele in these mice [22]. At 8M (~2 months following initial tamoxifen injection), *ORAI1* transcript levels in iORAI1-KO/GS mice were reduced by ~35% compared to ifl/GS controls (Supplemental Figure S2A). We then conducted *in vivo* behavioral and plethysmography assessments on tamoxifen-treated ifl/GS and iORAI1-KO/GS mice. Notably, 8M iORAI1-KO/GS mice were unable to complete the treadmill test (Supplemental Figure S2B), as observed for 4M cORAI1-KO/GS mice (Figure 2C). However, unlike 4M cORAI1-KO/GS mice, 8M iORAI1-KO/GS mice exhibited normal rotarod endurance, grip strength, and respiratory function (Supplemental Figure S2C–E).

## 4. Discussion

TAM is an autosomal dominant disorder, with only heterozygous presentation reported clinically. In line with this, we previously described a novel dominant mouse model of TAM (*ORAI1^G100S/+^* or ‘heterozygous’ GS mice) that results from a G100S pore mutation in a single *ORAI1* allele [42]. Heterozygous GS mice recapitulate several key clinical features of TAM in humans heterozygous for the analogous ORAI1-G98S variant, including muscle weakness, exercise intolerance, hypocalcemia, elevated CK levels, and the presence of TAs. Interestingly, intercrossing heterozygous GS mice fails to produce viable homozygous offspring or homozygous embryos [42]. Based on these findings, we conclude that GS homozygosity results in early embryonic lethality (<embryonic day 14) due to a critical role of ORAI1 in embryonic development. To evaluate the hypothesis that homotypic ORAI1-G100S expression in skeletal muscle leads to more severe phenotypes, we generated mice in which skeletal muscle contains only a single functional *G100S-ORAI1* allele (i.e., cORAI1-KO/GS or homotypic GS mice). Unlike homozygous (*ORAI1^G100S/G100S^*) mice, cORAI1-KO/GS are viable and survive into adulthood, presumably because homotypic GS expression is restricted to only skeletal muscle (i.e., all other tissues retain a functional *WT ORAI1* allele). Interestingly, similar to that reported previously for adult heterozygous GS mice [42], constitutive and store-operated Ca^2+^ entry are essentially abolished in FDB fibers from both 4M heterotypic GS (cfl/GS) and homotypic GS (cORAI1-KO/GS) mice (Figure 1D,E). Unexpectedly, we also found that constitutive, muscle-specific ablation of a single *ORAI1* allele (cORAI1-KO/+, cORAI1-KO/GS) also resulted in the near abolition of SOCE (Figure 1E) and a reduction in type IIx fiber CSA (Figure 5B), as previously reported for mice with constitutive, muscle-specific knockout of both *ORAI1* alleles [22].

What mechanisms might be responsible for the observed abolition of constitutive and store-operated Ca^2+^ entry in muscle fibers from both 4M heterotypic GS (cfl/GS) and homotypic GS (cORAI1-KO/GS) mice? Since constitutive Ca^2+^ entry would be expected to drive protease activation, myofiber damage, necrosis, and muscle regeneration, we speculate that compensatory mechanisms designed to limit uncontrolled trans-sarcolemmal Ca^2+^ influx are needed to protect muscle fibers from excessive Ca^2+^-mediated damage. Potential mechanisms that would limit constitutive Ca^2+^ entry include reduced ORAI1 expression, increased ORAI1 degradation, inhibition of ORAI1 activity through post-translational modifications [60], and mis-localization of ORAI1 channels to membrane compartments other than the surface membrane (e.g., endocytic vesicles or sequestration into TAs). Given that the *ORAI1* transcript level is reduced in muscle of homotypic GS mice (Supplemental Figure S2A) and ORAI1 protein expression is reduced in muscle of heterozygous GS mice, even in the absence of a change in the *ORAI1* transcript level [42], we expect that a reduction in ORAI1 protein expression also occurs in skeletal muscle of homotypic GS mice. Potential additional protective effects of inhibitory ORAI1 post-translational modifications or altered subcellular distribution of channels away from the surface membrane (although this was not observed in muscle of heterozygous GS mice) [42] are possible and remain to be evaluated.

**Figure 6:**
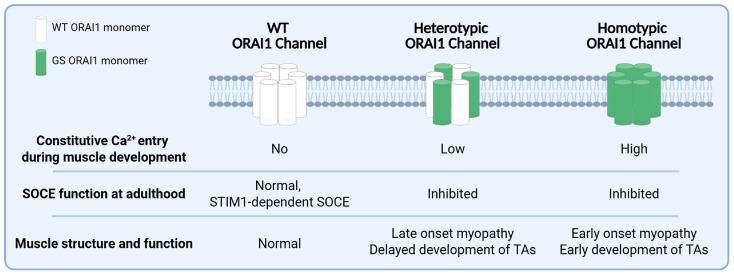
Summary of the main findings and predictions. ORAI1 hexameric channels can be comprised of only WT subunits (normal channels), heteromers of WT and GS subunits (heterotypic channels), or only GS subunits (homotypic GS channels) that exhibit distinct effects of ORAI1 channel function (i.e., constitutive- and store-operated calcium entry) and muscle structure/function during development and adulthood.

We found that the progression of skeletal muscle disease progression was accelerated in homotypic GS mice, in terms of both histological features and muscle performance (Figure 6). 

Unlike heterozygous GS mice in which TAs begin to appear at 8M [42], TAs were detected by Gomori Trichrome staining in homotypic GS mice as early as 4M (Supplemental Figure S1). In addition, homotypic GS mice exhibited significantly reduced *in vivo* muscle performance in grip strength, treadmill endurance, and rotarod endurance tests (Figure 2). In fact, most homotypic GS mice were unable to complete the rotarod endurance test, and none was able to finish the treadmill endurance assay, while all age-matched heterotypic GS (cfl/GS) mice completed both tests. Moreover, *ex vivo* muscle contractility measurements revealed a larger reduction in maximal specific force production in EDL muscles from homotypic GS mice compared that observed for WT (cfl/+) and heterotypic GS (cfl/GS) mice (Figure 4). These findings are consistent with homotypic expression of the G100S-ORAI1 mutation in skeletal muscle resulting in an earlier-onset and more severe muscle phenotype. The schematic in Figure 6 summarizes the key findings of this study and presents our working model regarding the importance of constitutive Ca^2+^ entry through G100S-ORAI1 channels during early muscle development in determining disease onset and severity.

Unlike heterozygous GS mice (and TAM patients), in which muscle weakness is predominantly localized to distal muscles, homotypic GS mice also exhibited respiratory dysfunction (Figure 3). Notably, homotypic GS mice exhibited significantly reduced breathing rate, mid-tidal expiratory flow, and peak inspiratory/expiratory flow compared to that of WT (cfl/+), heterotypic GS (cfl/GS), and cORAI1-KO/+ mice. These findings suggest that constitutive, homotypic GS expression in skeletal muscle results in a broader range of muscle dysfunction, impacting both limb and ventilatory function. However, as respiratory function reflects a complex interplay between multiple muscle groups, including diaphragm and intercostal muscles, additional targeted *ex vivo* studies are needed to determine the relative role of diaphragm versus intercostal muscles in the respiratory dysfunction observed in homotypic GS mice.

Interestingly, the skeletal-muscle phenotypes of inducible, muscle-specific homotypic GS (iORAI1-KO/GS) mice were less severe than those observed for constitutive, muscle-specific homotypic GS mice. Although inducible, homotypic GS mice exhibit markedly reduced exercise intolerance during the treadmill endurance test, other behavioral muscle performance measures (e.g., wire hang time, rotarod endurance and respiratory function) were unaltered (Supplemental Figure S2). The reason for the attenuated phenotype of inducible homotypic GS mice is unclear but could involve developmental and/or progressive effects on muscle function of constitutive homotypic GS expression in skeletal muscle. Alternatively, the milder phenotype of these mice could reflect an incomplete knockout of the *WT* allele (and protein). Consistent with this, while *ORAI1* mRNA was reduced ~50% in constitutive, homotypic GS mice (Figure 1A), *ORAI1* mRNA was reduced only ~35% in the inducible, homotypic GS mice (Supplemental Figure S2A).

## 5. Conclusions

In summary, results from this study demonstrate that homotypic GS expression exacerbates the TAM phenotype, as reflected in a more severe and earlier-onset phenotype than heterotypic GS mice. While the significance of this finding for TAM in humans is unclear, many inherited disorders are exacerbated when a disease-causing variant is co-inherited with a second hypomorphic allele for the same gene (as observed in RYR-1 myopathy) [61]. Thus, some cases of severe, early-onset TAM could result from co-inheritance of a dominant ORAI1 TAM variant together with a second hypomorphic ORAI1 allele (e.g., due to a nonsense variant, frameshift mutation, splice-site mutation, etc.). This possibility should be considered by neurologists and genetic counselors treating TAM patients that exhibit a more severe or earlier-onset presentation.

## Figures and Tables

**Table 1:** Summary of primer sequence used in genotyping.

**Cre** **Recombinase**	**Forward Sequence**	**5′-GCCTGCATTACCGGTCGATGCAACGA-3′**
	**Reverse Sequence**	5′-GTGGCAGATGGCGCGGCAACACCATT-3′
**Flox *ORAI1***	**Forward Sequence**	*ORAI1-flox-ko* 5′-TATGGTAAGGCTGGGAGACACT-3′ *ORAI1-flox* 5′–GGGACAAAACACTAACCTGTCAT–3′
	**Reverse Sequence**	5′-GGAGTAGAATTCAGTGGGAGAGT-3′

**Table 2:** Summary of primer sequence used in RT-qPCR.

**GAPDH**	**Forward Sequence**	5′-AGGAGAGTGTTTCCTCGTCC-3′
	**Reverse Sequence**	5′-TGAGGTCAATGAAGGGGTCG-3′
**ORAI1**	**Forward Sequence**	5′-GATCGGCCAGAGTTACTCCG-3′
	**Reverse Sequence**	5′-TGGGTAGTCATGGTCTGTGTC-3′

## Data Availability

The original contributions presented in this study are included in the article/Supplementary Materials. Further inquiries can be directed to the corresponding author.

## Supplementary Materials

The following supporting information can be downloaded at: https://www.mdpi.com/article/10.3390/biomedicines14030587/s1. Figure S1: Representative Gomori trichrome images of EDL muscles from 4M cfl/+, cfl/GS, cORAI1-KO/+, and cORAI1-KO/GS mice; Figure S2: Phenotype of iORAI1-KO/GS mice.
