# Supplementary figures and images for: Exacerbated Skeletal Muscle Phenotype in Mice with ‘Homotypic’ Expression of the Tubular Aggregate Myopathy ORAI1 G100S Mutation
Source: Biomedicines. 2026 Mar 5;14(3):587. doi: 10.3390/biomedicines14030587 (PMC13023914; doi:10.3390/biomedicines14030587)

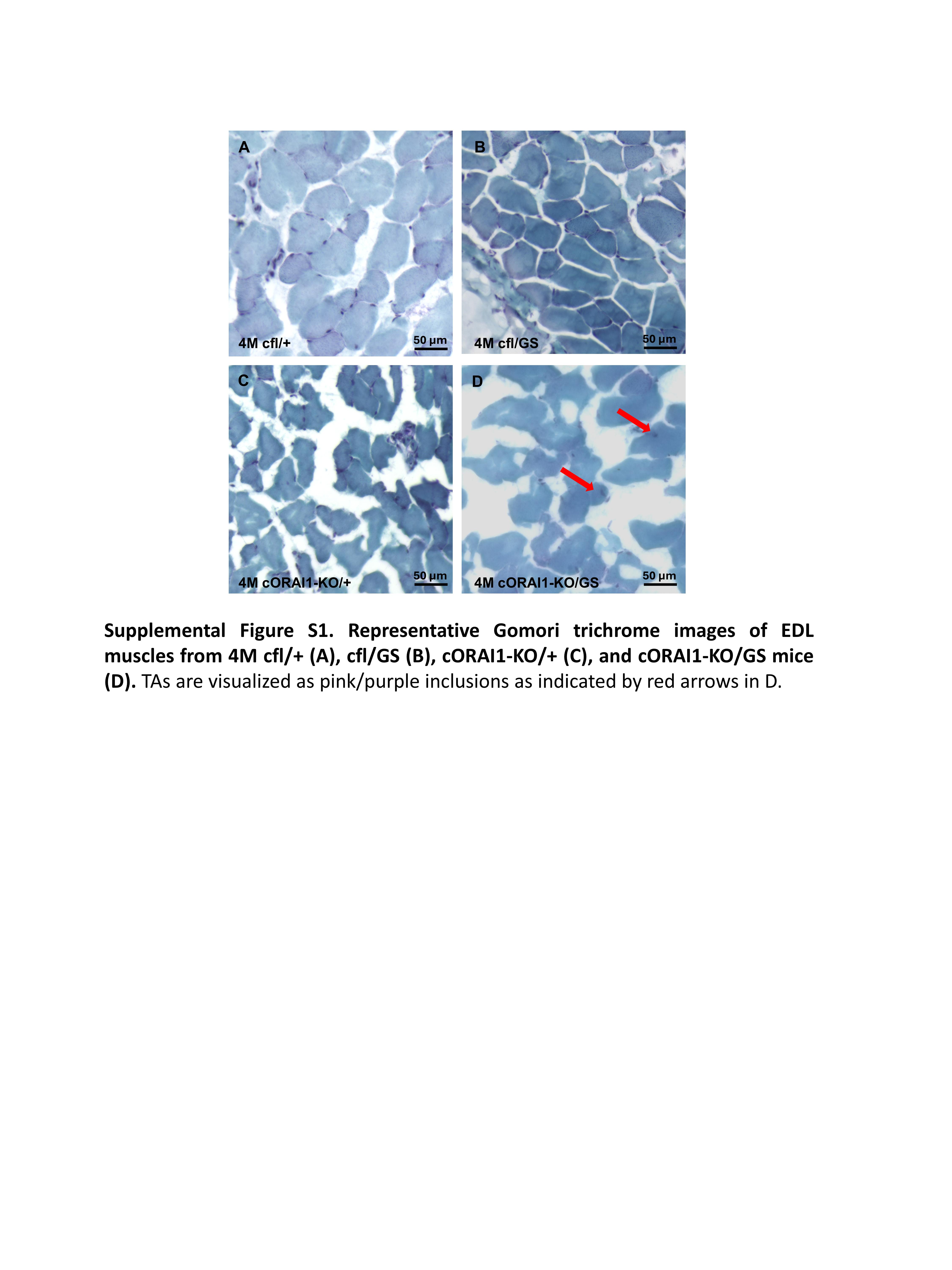

Supplement: Supplementary file 1 [file biomedicines-14-00587-s001.zip › Supplemental Figure 1 and Legend.tif]

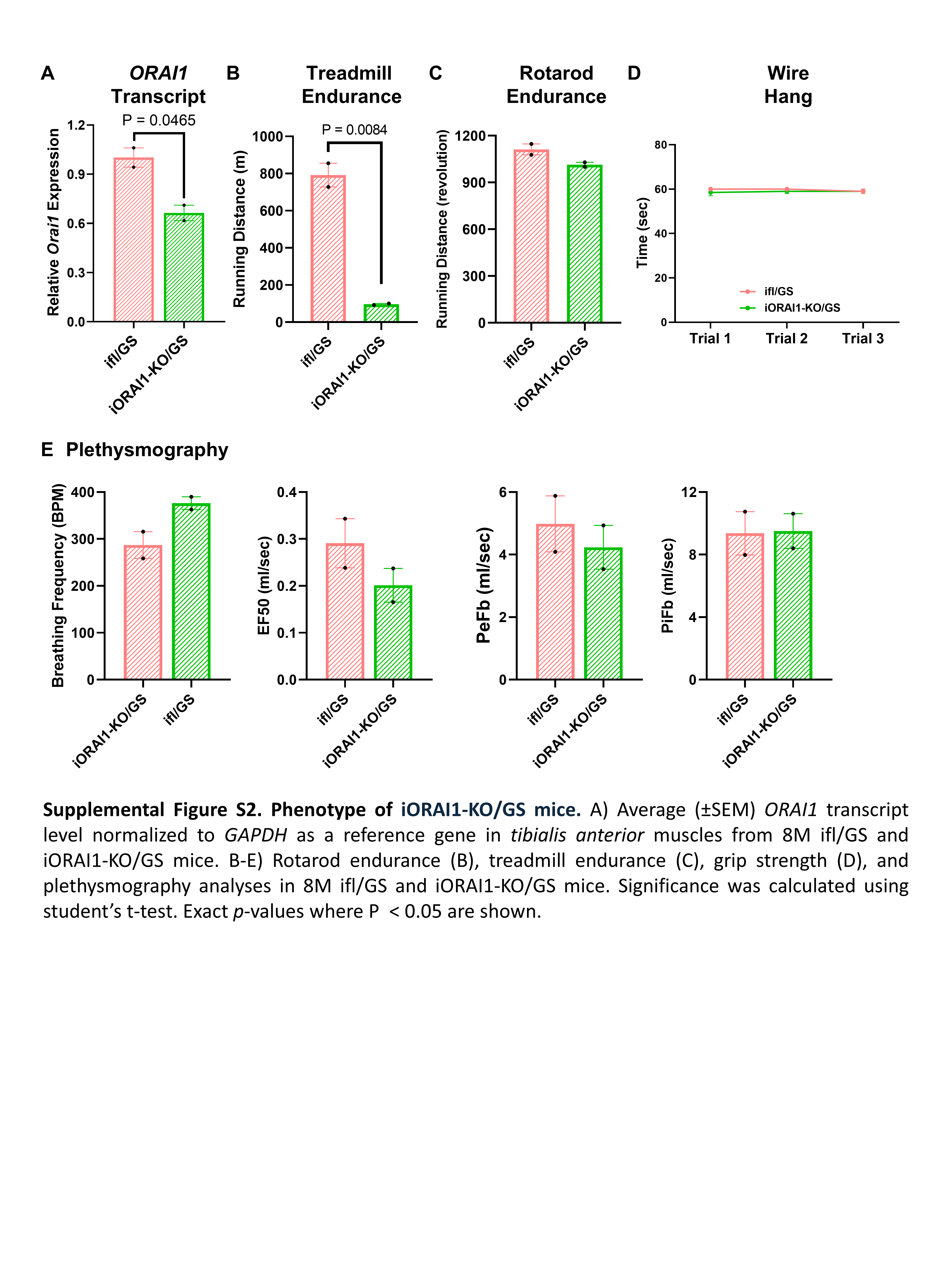

Supplement: Supplementary file 1 [file biomedicines-14-00587-s001.zip › Supplemental Figure 2 and Legend.tif]
